# Exploring a Novel Anti-Inflammatory Therapy for Diabetic Retinopathy Based on Glyco-Zeolitic-Imidazolate Frameworks

**DOI:** 10.3390/pharmaceutics17060791

**Published:** 2025-06-17

**Authors:** Elena Díaz-Paredes, Francisco Martín-Loro, Rocío Rodríguez-Marín, Laura Gómez-Jaramillo, Elena M. Sánchez-Fernández, Carolina Carrillo-Carrión, Ana I. Arroba

**Affiliations:** 1 Department of Endocrinology, INiBICA, Puerta del Mar University Hospital, University of Cádiz, Avda. Ana de Viya 21, 11009 Cádiz, Spain; elena.diaz@inibica.es (E.D.-P.); francisco.martin@inibica.es (F.M.-L.); laura.gomez@inibica.es (L.G.-J.); anaisabel.arroba@uca.es (A.I.A.); 2 Department of Organic Chemistry, Faculty of Chemistry, University of Sevilla, C/ Profesor García González 1, 41012 Sevilla, Spain; rrodriguez23@us.es; 3 Institute for Chemical Research (IIQ), CSIC—University of Seville, Avda. Américo Vespucio 49, 41092 Sevilla, Spain

**Keywords:** glycolipid, zeolitic-imidazolate framework, microglia, inflammation, immune-mediated therapy, diabetic retinopathy

## Abstract

**Background/Objectives:** Diabetic retinopathy is an ocular disease caused by changes in the expression of inflammatory mediators and increased oxidative stress in the retina and is the leading cause of vision loss in diabetic patients. Currently, there is no treatment capable of reversing retinal damage, which represents a significant burden on the quality of life of patients. (1*R*)-1-Dodecylsulfonyl-5*N*,6*O*-oxomethylidenenojirimycin stands outs as a prototype of the sp^2^-iminoglycolipids family for its beneficial neuroprotective effect against this chronic eye disease. Critical issues related to the low solubility and bioavailability of this glycolipid in biological settings are overcome by its encapsulation in a Zeolitic-Imidazolate Framework (ZIF) structure, resulting in homogeneous and biocompatible GlycoZIF nanoparticles. Cell studies show an enhanced cellular uptake compared with the free glycolipid, and importantly, its bioactivity is preserved once released inside cells. **Methods:** Extensive in vitro and ex vivo assays with diabetic retinopathy models unveil the mechanistic pathways of the designed GlycoZIF. **Results:** A reduction in proinflammatory mediators, increased heme oxygenase-1 level, inhibition of NLRP3 inflammasome, and reduced reactive gliosis is shown. **Conclusions:** These findings demonstrate for the first time the potential of Glyco-modified ZIFs for the treatment of diabetes-related ocular problems by controlling the immune-mediated inflammatory response.

## 1. Introduction

Diabetic retinopathy (DR), one of the primary diabetes-related complications, is considered a chronic inflammatory disease that may trigger major ocular problems in patients suffering from diabetes mellitus (DM). Food and Drug Administration (FDA)-approved treatments against this pathology are based on targeting upregulated vascular and inflammatory mediators in the retina [1]. Recurrent intravitreal injections of vascular endothelial growth factor inhibitors (anti-VEGFs) such as bevacizumab (Avastin^®^), ranibizumab (Lucentis^®^), and aflibercept (Eylea^®^) or anti-inflammatory corticosteroids such as dexamethasone (Ozurdex) stand out among the first-line drugs aimed at mitigating vision loss or slowing the progression of this multifactorial eye disease [2,3]. However, this therapeutic approach entails some drawbacks, such as the high cost of these treatments, the probability of vitreous hemorrhages, the systemic toxicity, the poor response of some patients, and the stress caused by frequent intravitreal injections, thus complicating DR management. Considering the increasing prevalence of diabetes globally [4], estimated to be 10.2% (578 million people) by 2030 and 10.9% (700 million) by 2045 [5], it is an urgent need to search for more effective, safer, and less invasive immunomodulatory therapeutic approaches targeting inflammation and be able to reverse retinal lesions to restore (at least partially) visual acuity. These figures clearly indicate that DR management should be established as a priority research area due to its relevance to current and future public health.

**Figure 1:**
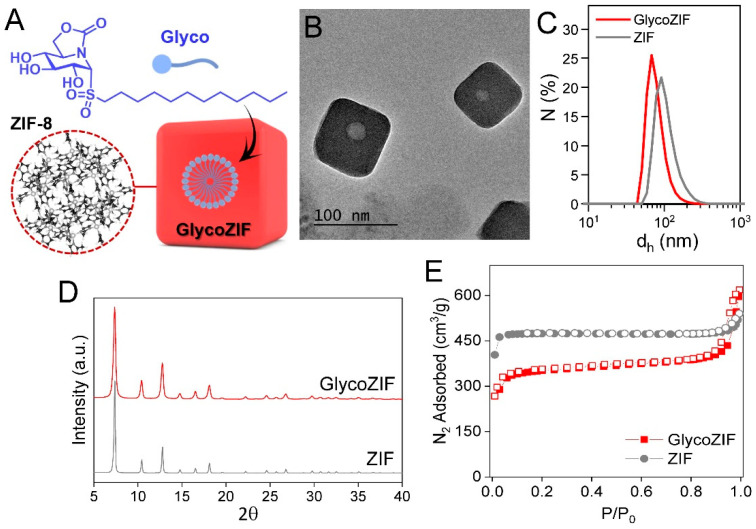
(**A**) Chemical structure of the glycolipid studied (Glyco) and scheme of a GlycoZIF particle containing glycolipid molecules encapsulated in the form of micelles. (**B**) TEM image of the GlycoZIF particles showing the Glyco-micelle inside. (**C**) DLS number distributions of the GlycoZIF and control ZIF particles dispersed in Milli-Q water. (**D**) PXRD patterns of the GlycoZIF and ZIF particles. (**E**) N_2_ isotherms (77 K) of the GlycoZIF and ZIF particles. Filled symbols represent adsorption, while empty ones represent desorption.

On this basis, the sp^2^-iminoglycolipids (sp^2^-IGLs), a highly versatile family of stable glycoconjugate mimetics, have attracted special attention as drug candidates [6] for their ability to act as immune system response regulators with antiproliferative [7], antiparasitic [8], and anti-inflammatory [9,10] therapeutic properties against a broad range of pathologies. Specifically, (1*R*)-1-dodecylsulfonyl-5*N*,6*O*-oxomethylidenenojirimycin (DSO_2_-ONJ, Figure 1A) is highlighted within this sp^2^-IGLs-based family as a functional immunomodulatory prototype in the context of DR that is able to restrain inflammation and reduce reactive gliosis [11]. Our previous data, resulting from both in vitro (murine Bv.2 microglial cells) and ex vivo (retinal explants from diabetic mice) experimental assays, have revealed the neuroprotective effect of this nojirimycin (NJ)-related derivative able to antagonize, in the micromolar range (10–20 µM), the expression levels of some proinflammatory cytokines and induce an anti-inflammatory response mediated by autophosphorylation of p38α MAPK (mitogen-activated protein kinase) [11].

Despite such interesting results, these sp^2^-IGLs have limited solubility in aqueous biological media, resulting in a low bioavailability and reduced cell membrane permeability when administrated as free therapeutic agents in in vitro and ex vivo systems. In the particular case of ocular disease management, several drug delivery systems (DDSs) have been developed already (e.g., nanomicelles, nanoparticles, nanosuspensions, nanoemulsions, liposomes, dendrimers) for treating dry eye disease, macular edema, or ocular bacterial infections [12]. In general terms, all these DDSs pursue the following benefits: (i) decreased intrinsic drug toxicity and degradation, (ii) enhanced drug permeability to cross ocular biological barriers, (iii) extended drug residence time, and (iv) modulated release kinetics to provide optimal therapeutic concentrations, minimizing side effects in healthy tissues. However, there is no an ideal nanosystem that meets all these advantages at the same time, and for this reason, many research efforts continue to focus on the design of new or improved nanoplatforms.

In this line, Metal–Organic Frameworks (MOFs), a type of crystalline porous materials constituted by polydentate organic ligands and inorganic centers, have emerged as promising DDSs to treat retina-related disorders [13]. Indeed, the encapsulation of available anti-inflammatory drugs with ophthalmic uses (e.g., brimonidine, methylprednisolone, and dexamethasone) in MOFs has been evaluated against ocular complications such as chronic glaucoma, photoreceptor degeneration, and keratitis, resulting in improved results regarding toxicity, drug dosage, and long-term drug release in in vitro and in vivo models [14,15]. However, these reported results are mainly based on the observed cellular activities, but with little or no knowledge of the mechanisms behind the obtained improved effectiveness. In-depth studies of these mechanistic aspects would be very beneficial, since they would allow a more rational design of these DDSs to precisely modulate their performance and outcomes. Currently, most nanosystem-based therapeutic strategies in the context of DR focus on preventing disease progression [16], while an early-stage approach to the inflammatory events that characterize the onset of DR would be much more promising, as it would help to inhibit the development of irreversible processes such as neurodegeneration and loss of visual function [17].

Based on these premises, the main goal of this study is to evaluate the immunomodulatory potential of a GlycoZIF nanosystem as a novel therapeutic strategy for early-stage DR. Rather than focusing on the encapsulation process itself [18,19], our work aims to assess the biological efficacy of a GlycoZIF, which incorporates the anti-inflammatory agent DSO_2_-ONJ (Figure 1A), in modulating inflammation in relevant in vitro and ex vivo diabetic models. Murine Bv.2 microglial cells (retinal immune cells) and retinal explants from BB rats (an animal model of human type 1 DM (T1DM)) were used as in vitro and ex vivo diabetic-inflammatory models, respectively, to evaluate cellular uptake, anti-inflammatory effects, and mechanistic pathways. Particular attention was paid to the modulation of the key inflammatory mediators and pathways involved in the immune-mediated inflammatory response.

## 2. Materials and Methods

### 2.1. Chemicals and Materials

All reagents were obtained from commercial sources and were used without further purification. Zinc nitrate hexahydrate (Zn(NO_3_)_2_·6H_2_O), 2-methylimidazole (HmIM), and hexadecyltrimethylammonium bromide (CTAB) were obtained from Sigma-Aldrich. Thin-layer chromatography was performed on precoated TLC plates, silica gel 30F-245, with visualization by UV light and by carrying with 10% H_2_SO_4_ or 0.2% *w*/*v* cerium (IV) sulfate-5% ammonium molybdate in 2 m H_2_SO_4_ or 0.1% ninhydrin in EtOH. Column chromatography was performed on Chromagel (silice 60 AC.C 70–200 μm). Fetal bovine serum (FBS) and culture media were obtained from Invitrogen. RPMI 1640 medium, bovine serum albumin (BSA), Crystal Violet, glutaraldehyde, *N*-(1-naphthyl)ethylenediamine (NEDA), sulfanilamide, Triton X-100, sucrose, bacterial lipopolysaccharide (LPS), sodium dodecyl sulfate (SDS), penicillin/streptomycin, and DL-dithiothreitol (DTT) were purchased from Sigma-Aldrich (Merck, Mollet del Vallès Barcelona, Spain). Protease inhibitors complete-EDTA free were obtained from Roche and Accutase from BioLegend. Acrylamide and immunoblot PVDF membranes were purchased from Bio-Rad. The Bicinchoninic Acid (BCA) protein assay and cell culture inserts (pore size 0.4 μm) were purchased from Thermo Fisher (Waltham, MA, USA), and Fluoromount-G was obtained from Southern Biotech (Birmingham, AL, USA). Thiobarbital was obtained from Braun Medical and L-glutamine from Gibco (Waltham, MA, USA). LysoTracker^®^ was obtained from Molecular Probes, Thermo Fisher (Waltham, MA, USA). Antibodies used are described in Table S1. Rat primers for transcripts *Il6*, *Il1b*, *Tnfa*, and *Gapdh* (Table S2) were purchased from Applied Biosystems (Waltham, MA, USA).

### 2.2. Instrumentation for Physicochemical Characterization

^1^H (^13^C) Nuclear Magnetic Resonance Spectroscopy (NMR) spectra were performed on Bruker Avance spectrometers at 300, 400, and 500 MHz (75.5, 100.6, and 125.7). High-performance liquid chromatography (HPLC) analysis was carried out using a Waters Alliance 2695 HPLC (Waters Corporation, Millford, DE, USA) coupled to an ESI-ion trap mass spectrometer instrument (Bruker AmaZon, Karlsruhe, Germany). Samples were analyzed using 0.1% formic acid eluting gradients at a flow rate of 0.3 mL/min. Transmission Electron Microscopy (TEM) images were acquired using a JEOL 2100Plus (JEOL USA, Inc., Peabody, MA, USA) operated at 200 kV. Dynamic Light Scattering (DLS) measurements were performed on a Malvern Zetasizer Nano (Malvern Instruments, Westborough, MA, USA) equipped with a 10 mW He–Ne laser operating at 633 nm and a scattering angle of 173°. Powder X-ray Diffraction (PXRD) was performed using a Bruker D8-Advance Diffractometer (Bruker AXS GmbH, Karlsruhe, Germany) with X-ray radiation of Cu Kα. N_2_ physisorption analysis was carried out at 77 K on a Micromeritics Tristar II 3020 system (Micromeritics, Norcross, GA, USA). Samples were degassed for 18 h at 120 °C under vacuum. The surface area was calculated from the Brunauer–Emmett–Teller (BET) equation, and pore volume and external surface area were determined by t-plot method.

### 2.3. Synthetic Procedures

#### 2.3.1. Synthesis of the Glycolipid

(1*R*)-1-Dodecylsulfonyl-5*N*,6*O*-oxomethylidenenojirimycin (DSO_2_-ONJ) was synthesized by oxidation of its precursor (1*R*)-2,3,4-tri-*O*-acetyl-1-dodecylthio-5*N*,6*O*-oxomethylidenenojirimycin with an excess of *m*-chloroperbenzoic acid and deprotection reaction of the *O*-acetyl groups under Zemplén conditions following the procedures previously reported [11].

#### 2.3.2. Preparation of the GlycoZIF and ZIF Control Particles

Synthesis was carried out by following our reported optimized protocols [18,19]. Briefly, an aqueous solution of Zn(NO_3_)_2_ (3 mL, 0.025 m) was added to an aqueous solution of 2-methylimidazole (HmIM; 3 mL, 1.3 m) at room temperature under stirring (350 rpm). Afterwards, a methanolic solution of Glyco (0.6 mL, 10 mM) was added dropwise to the mixture, stirred for 2 min, and allowed to react for 2 h under static conditions. The resulting particles were purified by centrifugation (13,000 rpm, 15 min), washed three times with methanol (MeOH), and finally dispersed in MeOH at 10 mg/mL. ZIF-8 nanoparticles were prepared as the control sample by following the same procedure but replacing the amphiphilic glycolipid with the surfactant CTAB (3 mL, 0.002 mM) as size-controlling agent.

Fluorescent-labeled GlycoZIF nanoparticles were prepared to monitor the cell uptake by flow cytometry and confocal microscopy. For that, the as-synthetized GlycoZIF nanoparticles, as dispersed in MeOH (0.2 mL, 10 mg/mL), were incubated with a methanolic solution of fluoresceinamine (FA, 100 µL, 1 mg/mL) at room temperature for 18 h. Afterwards, the excess of the dye was removed by centrifugation, the FA@GlycoZIF particles were washed twice with MeOH, and finally dispersed in MeOH at 10 mg/mL. The same procedure was carried out to prepare the fluorescent-labeled ZIF control nanoparticles (FA@ZIF).

The encapsulation efficiency (EE%) was determined by HPLC quantification of the glycolipid remaining in the supernatant (i.e., *Glyco*_non-encapsulated_) after centrifugation and washing steps of the GlycoZIF particles, and knowing the amount of Glyco added initially (i.e., *Glyco*_added_), applying the following equation:

$$\begin{array}{l} EE(\%)=\frac{{\mu mol\;Glyco}_{encapsulated}}{{\mu mol\;Glyco}_{added}}\times 100\\ \;=\frac{{\mu mol\;Glyco}_{added}{\;-\;\mu mol\;Glyco}_{non-encapsulated}}{{\mu mol\;Glyco}_{added}}\times 100 \end{array}$$

The loading capacity (LC%) was calculated by dissolving the GlycoZIF particles (50 μL at 10 mg/mL) with HCl (20 μL) and then quantifying the amount of Glyco encapsulated (i.e., *Glyco*_encapsulated_) by HPLC analysis. The following procedure was used: stock solution (10 mg/mL) was incubated with 20 μL of HCl to destroy (dissolve) the particles, and the resulting mixture was analyzed by HPLC-MS. In this way, the amount of Glyco encapsulated per gram of particles was calculated using the following equation:

$$LC\;\left(\mathrm{wt}\%\right)=\frac{{mg\;Glyco}_{encapsulated}}{mg\;GlycoZIF}\times 100$$

### 2.4. In Vitro and Ex Vivo Protocols

#### 2.4.1. Cell Cultures

The murine Bv.2 microglial cell line was purchased from ACCEGEN Biotechnology. A total of 1.5 × 10^5^ cells were seeded per well in a 6-well plate. The cells were cultured at 37 °C in a humidified atmosphere with 5% CO_2_ in RPMI medium supplemented with 10% (*v*/*v*) heat-inactivated FBS, 1% (*v*/*v*) penicillin/streptomycin, and 2 mM L-glutamine. Bv.2 cells between passages 10–20 were used for experiments. Bv.2 cells were grown up to 70% confluence, washed with PBS, and further treated in serum-free medium with GlycoZIF or ZIF (500 nM) for 24 h (optimized incubation time) or in Glyco for 3 h (optimized incubation time), followed by LPS stimulation (200 ng/mL) for another 24 h.

#### 2.4.2. Cell Viability

Cells were cultured in serum-free media and treated with Glyco, ZIF, and GlycoZIF at different concentrations (0.1 μM, 0.5 μM, 1 μM, 10 μM) for 48 h to assess the cellular viability using Crystal Violet staining. Untreated cells were used as control cells. After treatments, the media were discarded and the remaining viable adherent cells were fixed with 10% glutaraldehyde and stained with Crystal Violet (0.1% *w*/*v* in water) for 20 min. The plates were then rinsed with tap water and allowed to dry. Acetic acid (10%) was added to solubilize the Crystal Violet. The absorbance of each plate was read spectrophotometrically at 590 nm on a microplate reader spectrophotometer (BioTek PowerWave, Bioteck, Torino, Italy). Cell viability is expressed as fold-change values relative to untreated control cells (viability of control cells was set as 1).

#### 2.4.3. Cell Internalization of GlycoZIF and ZIF Particles

GlycoZIF and ZIF labeled with fluoresceinamine (FA@GlycoZIF and FA@ZIF) were used to study their cellular uptake. For that, Bv.2 cells were treated with FA@GlycoZIF or FA@ZIF (500 nM) for different times (0 h, 3 h, 24 h, and 48 h). Following the incubation, cells were washed with PBS and detached with trypsin for 5 min at 37 °C. After cell harvesting, they were centrifuged (2000 rpm, 5 min), washed with PBS, and diluted in 400 µL of PBS. The resuspended cells were stored on ice and protected from light until flow cytometric analysis. The samples were analyzed using a FACSCelesta SORP (BD biosciences). FA@ZIF and FA@GlycoZIF fluorescence was excited at 633 nm, and the emission was recorded using a 661/16 nm bandpass filter. Post-acquisition, the results were analyzed using the FlowJo software (v10.8). Experiments were conducted in triplicate, wherein 1000 cells were measured for each experiment, and results were presented as the mean fluorescence intensity (MFI).

For the investigation of the intracellular location of the GlycoZIF nanoparticles after internalization, experiments with LysoTracker staining were additionally performed using confocal microscope Axiovert (Zeiss, Jena, Germany). A total of 20,000 cells per well were seeded in an 8-well Ibidi slide and incubated with GlycoZIF or ZIF particles (500 nM) following the identical experimental protocol described above. For staining, cells were incubated with LysoTracker staining solution in a concentration of 75 nM for 30 min at 37 °C. Afterwards, the staining solution was aspirated and the cells were washed twice with 250 μL of fresh medium. Finally, 250 μL/well was added, and the living cells were immediately analyzed by confocal scanning microscopy.

#### 2.4.4. Analysis of Nitrites (NO_2_^−^)

Only Bv.2 microglial cells are able to produce nitrites under a proinflammatory stimulus. To determine the nitrites production through the Griess test [20], the cells were treated with ZIF or GlycoZIF (500 nM) for 24 h or Glyco (500 nM) for 3 h and stimulated with LPS (200 ng/mL) for another 24 h, which mimics the diabetic proinflammatory environment [11]. Briefly, nitrites turn a pink color in contact with an acid solution containing 1% sulfanilamide and 0.1% NEDA. The production of nitrites was quantified spectrophotometrically at 540 nm in a microplate reader. Nitrite production is expressed as fold-change values relative to untreated control cells (nitrite production of control cells was set as 1).

#### 2.4.5. Quantitative Real-Time Polymerase Chain Reaction (qRT-PCR) Analysis

Total RNA was extracted with Trizol^®^ reagent (Invitrogen, Madrid, Spain) and reverse transcribed using an iScript™ gDNA clear cDNA Synthesis kit (BioRad, Hercules, CA, USA) for qPCR following the manufacturer’s recommendations. qRT-PCRs were performed in a CFX96 Touch™ (BioRad, Hercules, CA, USA) detection system from Bio-Rad laboratories.

#### 2.4.6. Western Blot Analysis

Equal amounts of proteins (20–40 μg) were resolved using denaturing SDS-PAGE and transferred to PVDF membranes (Bio-Rad, Hercules, CA, USA). Membranes were blocked using 5% skim milk or 3% BSA in PBS (10 mM Tris-HCl, 150 mM NaCl, pH 7.5), and incubated overnight at 4 °C with primary antibodies (1:1000 unless otherwise stated) in T-PBS (0.05% Tween-20-PBS). Next, membranes were washed with T-PBS and incubated with the corresponding secondary peroxidase-conjugated antibody (1:2000) in blocking buffer for 2 h at room temperature. Blots were again washed with T-PBS, and the immunoreactive bands were visualized using the Western-Bright Sirius reagent from Advansta Inc and a ChemiDoc™ Imaging System (Bio-Rad, Hercules, CA, USA). Western blot quantification was performed using the ImageJ (1.52) program.

#### 2.4.7. Retinal Explant Cultures

Bio-Breeding (BB) and Wistar rats were maintained under conventional conditions in an environment-controlled room (20–21 °C, 12 h light–dark cycle) with water and standard laboratory rat chow available ad libitum. Blood samples from the tail vein were used in BB rats for weekly random glucose measurements using an automatic glucose monitor (Freestyle Optium Neo, Abbott, Madrid, Spain). Diabetes onset was defined by glucose levels above 270 mg/dL (14.98 mmol/L). Ex vivo assays were performed with retinas from 7-week-old male or female BB rats. The rats were euthanized by an overdose of anesthesia, and the eyes were enucleated. The lens, anterior segment, vitreous body, retinal pigment epithelium, and sclera were removed. The retinas were immediately cultured in R16 media with no additional serum on cell culture inserts with a pore size of 0.4 μm. Retinas were cultured with or without GlycoZIF (500 nM) for 24 h.

#### 2.4.8. Retinal Explants Immunofluorescence Analysis

The whole retinas were fixed in 4% (*w*/*v*) paraformaldehyde for 24 h at 4 °C. Next, they were washed in PBS containing 0.1% (*w*/*v*) BSA and 0.1% (*v*/*v*) Triton X-100 (TBS) and blocked and permeated for 2 h in TBS containing 3% (*w*/*v*) BSA and 1% (*v*/*v*) Triton X-100. Subsequently, the retinal explants were incubated with a rabbit anti-GFAP or anti-IBA-1 antibody in blocking solution (1:500) overnight in a humid chamber at 4 °C. Retinal sections and retinal explants were washed with TBS buffer and incubated for 90 min with anti-rabbit immunoglobulin antibody conjugated to AlexaFluor 488 (1:1000) ThermoFisher (Waltham, MA, USA). After washing, retinal explants were mounted on slides with Fluoromount G media containing DAPI for staining and were analyzed with an inverted laser confocal microscope Axiovert (Zeiss, Jena, Germany).

#### 2.4.9. Statistical Analysis

Values in all graphs are presented as mean ± standard deviation (SD). Statistical tests were performed using GraphPad Prism7.0a software. Data were analyzed using one-way ANOVA followed by Bonferroni test or Student’s paired t-test when comparisons were between any two groups. A *p*-value < 0.05 was considered statistically significant.

## 3. Results

### 3.1. Physicochemical Characterization of GlycoZIF Nanoparticles

Highly homogeneous GlycoZIF nanoparticles, consisting of a ZIF-8 nanostructure having encapsulated the Glyco anti-inflammatory agent (Figure 1A), were successfully prepared by following a synthetic strategy previously optimized by our group [18]. Note that GlycoZIF, which refers to ZIF-8 containing the DSO_2_-ONJ glycolipid, is used throughout the manuscript for simplicity. The encapsulation efficiency (EE) and loading capacity (LC) were 98% and 3.8 wt%, respectively, as determined by high-performance liquid chromatography (HPLC) analyses. The as-prepared GlycoZIFs presented a rounded cubic shape with a lateral size of ca. 70 nm, as determined by TEM (Figure 1B), and a hydrodynamic diameter (d_h_) of 78 ± 1.5 nm in water, as revealed by DLS measurements (Figure 1C). Notably, the population of GlycoZIF particles was very homogeneous, having a low polydispersity index (PDI = 0.16). The control ZIF particles presented a similar morphology and size (ca. 100 nm derived from TEM and 110 ± 3.5 nm of d_h_, Figure S1 and Figure 1C, respectively). PXRD revealed that the GlycoZIFs were highly crystalline, showing the same pattern as that obtained for the control ZIF nanoparticles, with all the diffraction peaks corresponding to the sodalite topology, as expected (Figure 1D). Regarding the porosity, N_2_ isotherms of the GlycoZIF showed a significant decrease in the micropore area along with an increase in the external surface compared with the control ZIF particles (Table S3), indicative of the incorporation of the Glyco molecules within the framework. ^1^H NMR also confirmed the incorporation of the glycolipid within the ZIF-8 structure and served to check that the Glyco compound preserved its structure once released after dissolution of the GlycoZIF particles (Figure S2). Together, these complementary techniques consistently validate the formation of structurally robust GlycoZIF nanoparticles with effective encapsulation of the glycolipid cargo. On the other hand, all physicochemical analyses performed on GlycoZIF nanoparticles obtained from independently prepared batches over time revealed consistent and reproducible results, confirming the high reliability and reproducibility of the synthetic protocol.

### 3.2. Cytotoxicity and Cellular Uptake of GlycoZIF Nanoparticles

**Figure 2:**
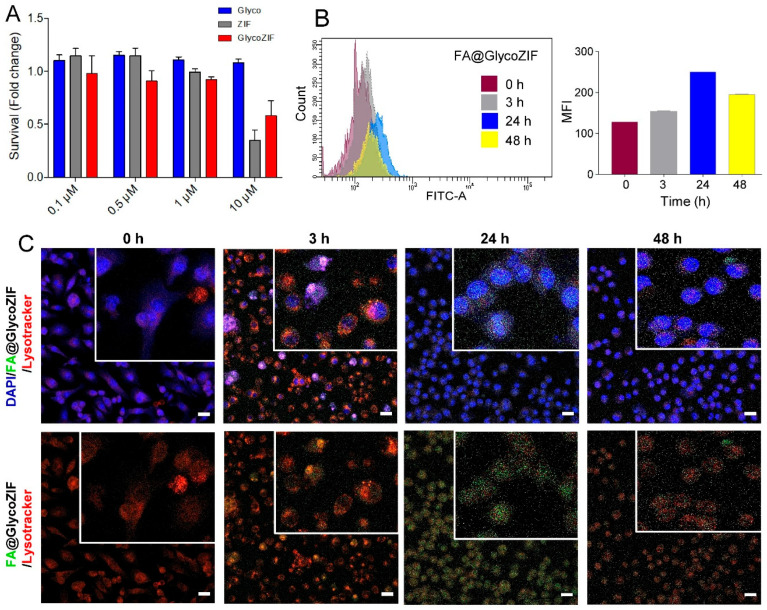
(**A**) Cell viability of Bv.2 microglial cells under 48 h exposure to increasing concentrations of Glyco, ZIF, or GlycoZIF (from 0.1 to 10 μM) as determined by Crystal Violet assay. Data are presented as fold-change values relative to untreated control cells. (**B**) Flow cytometric analysis of the uptake of FA@GlycoZIF (500 nM) by Bv.2 cells showing the histograms and the corresponding MFI values as a function of the exposure time (0 h, 3 h, 24 h, and 48 h). (**C**) Confocal microscopy images of Bv.2 cells incubated with FA@GlycoZIF (500 nM) for different exposure times (0 h, 3 h, 24 h, and 48 h). Green fluorescence corresponds to the encapsulated cargo (i.e., FA), while red fluorescence corresponds to LysoTracker and blue fluorescence to the stained nucleus with DAPI. Higher magnifications (white squares) are shown in the inset top right. Scale bar: 20 μm.

Firstly, we evaluated the cytotoxicity profile of the GlycoZIF nanosystem in our in vitro model of murine retinal immune cells to properly select an optimal concentration. For that, Bv.2 microglial cells were treated with different doses (from 0.1 µM to 10 µM) of GlycoZIF particles for 48 h, and the viable cells were measured by Crystal Violet assay. Glyco and ZIF particles were also studied as control samples. As shown in Figure 2A, we did not observe any cytotoxicity in the low μM range (from 0.1 µM to 1 µM), whereas at a higher concentration (10 µM), the cell viability decreased notably in the case of the GlycoZIF and ZIF particles. Based on that, the maximum concentration used for the following cell studies was fixed at 500 nM, to ensure a cell viability greater than 80% in all cases. It is worth noting that independently prepared batches of GlycoZIFs resulted in comparable cell viability profiles, further confirming the reproducibility in the preparation of these nanoparticles.

Next, we investigated the cellular uptake efficiency and the intracellular location of the GlycoZIF nanoparticles by flow cytometry and confocal microscopy, respectively. To be able to monitor the particles inside cells, the GlycoZIF was labeled with a green fluorescent probe, specifically fluoresceinamine (FA@GlycoZIF). The control ZIF particles were also labeled (FA@ZIF) and studied for comparison. Bv.2 cells treated with 500 nM of either FA@GlycoZIF or FA@ZIF were analyzed by flow cytometry after different times (0 h, 3 h, 24 h, 48 h), showing in both cases rather fast uptake kinetics, since a significant mean fluorescence signal per cell (MFI) was already found after 3 h of incubation time (Figure 2B). As expected, the uptake efficiency was time dependent, with intracellular fluorescence increasing as the incubation time increased. The maximum cell internalization for GlycoZIF was obtained after 24 h, whereas longer times led to a reduction in the intracellular fluorescence. This finding suggests the exocytosis rate of the fluorescent cargo (already released to the cytosol after 48 h) is faster than the endocytosis rate of GlycoZIF particles. The control ZIF particles presented a slightly slower uptake, as shown in Figure S3A. This difference in the uptake kinetics for GlycoZIF and ZIF particles could be attributed to their particle size, as the GlycoZIF nanoparticles were noticeably smaller (i.e., 78 nm of hydrodynamic diameter compared with 110 nm for ZIF, Figure 1C). This trend of uptake efficiency decreasing with an increase in the particle size of the MOF has been previously reported [21]. Based on these results, we selected 24 h as the optimal exposure time for the treatment of cells with GlycoZIF particles and for ZIF as the control sample. Confocal microscopy images (Figure 2C) revealed that at short times (3 h), the FA@GlycoZIF particles were localized mainly in subcellular organelles (endosomes/lysosomes), observing a high colocalization degree between the green fluorescent cargo (FA) and the red fluorescent LysoTracker probe. After 24 h, the green fluorescence was mainly present in the cytoplasm, and colocalization was lost as a result of the dissolution of the GlycoZIF particles at the acidic pH inside the endosomes/lysosomes and the subsequent release of the cargo to the cytosol. Similar results were obtained for the labeled FA@ZIF particles (Figure S3B).

### 3.3. Protective Effect of GlycoZIF Against LPS-Induced Proinflammatory Mediators on Bv.2 Microglial Cells

**Figure 3:**
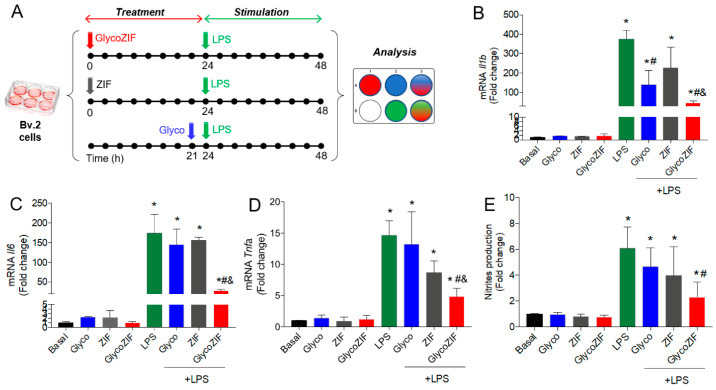
(**A**) Scheme of the in vitro protocol for optimal treatment with Glyco, ZIF, or GlycoZIF and further LPS stimulation for induction of inflammation in Bv.2 microglial cells. Cells were pretreated with ZIF or GlycoZIF (500 nM) for 24 h or with Glyco (500 nM) for 3 h and then further stimulated with LPS (200 ng/mL) for another 24 h. Quantification of (**B**) *Il1b*, (**C**) *Il6*, and (**D**) *Tnfa* mRNA levels determined by qRT-PCR. (**E**) Nitrites accumulation determined by the Griess method. Data were normalized to *Gapdh* gene expression. Results are expressed as mean ± SD (n = 5 independent experiments) and presented as fold-change values relative to untreated control cells (basal value). * *p* ≤ 0.05 vs. basal treatment, ^#^ *p* ≤ 0.05 vs. LPS treatment, ^&^ *p* ≤ 0.05 vs. Glyco + LPS treatment.

Once the non-cytotoxic dose range of the GlycoZIF was established and the efficient internalization in Bv.2 microglial cells was confirmed, our efforts focused on determining whether the GlycoZIF presented an effective activity at nanomolar concentrations under an inflammatory environment. Following the optimized experimental protocol depicted in Figure 3A, we studied the effect of GlycoZIF nanoparticles on two inflammatory mediators, IL-1β and IL-6, secreted after stimulation by LPS. These proinflammatory cytokines are well known to play a critical role in inflammation-related disorders [22]. The inhibitory effect on *Il1b* and *Il6* mRNA expression levels was significant at 500 nM concentration of GlycoZIF, whereas lower concentrations (50–100 nM) did not exert any favorable effect on the LPS-induced detrimental inflammatory environment, thus showing a dose-dependent response (Figure S4A,B). Note that the treatment with the free Glyco compound was of only 3 h, compared with 24 h for GlycoZIF and ZIF particles, because this time was the optimal incubation time for achieving a maximum anti-inflammatory activity of the Glyco as previously optimized [11]. Considering these results, 500 nM was selected as the optimal concentration in terms of both safety and potency for the following biological assays. Indeed, significant increases in LPS-induced *Tnfa* mRNA expression levels and nitrites production were greatly ameliorated in the presence of GlycoZIF at 500 nM, showing a 0.57- and 0.39-fold decrease compared with Glyco effects for *Tnfa* and nitrites, respectively (Figure 3B–E). It is worth noting that the free Glyco compound did not present any effect at such low concentration, clearly demonstrating the improvement in the cellular internalization of the glycodrug when encapsulated within the ZIF carrier. Notably, ZIF control particles also led to a 0.40- and 0.34-fold reduction in *Tnfa* and nitrites, respectively, revealing the intrinsic anti-inflammatory properties of the ZIF-8. These findings thereby reveal that the effect observed with GlycoZIF is additive or cumulative, leveraging the beneficial properties of both constituents within the GlycoZIF particles.

**Figure 4:**
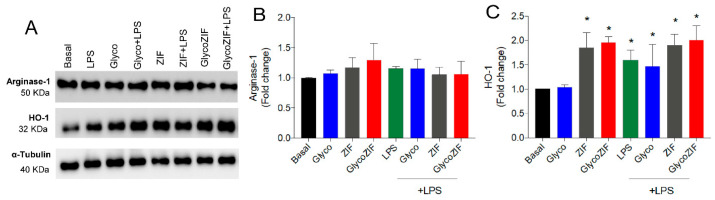
(**A**) Western blot analyses of proteins extracted from Bv.2 microglial cells subjected to the treatment protocol depicted in Figure 3A. Quantification of (**B**) Arginase-1 and (**C**) HO-1 levels. α-Tubulin was used as a loading control. Results are expressed as mean ± SD (n = 4 independent experiments) and presented as fold-change values relative to untreated control cells (basal value). * *p* ≤ 0.05 vs. basal treatment.

To better understand the potential effects of GlycoZIF as an inducer of the anti-inflammatory response, the changes in the enzymes Arginase-1, a straightforward marker of the M2 polarization state of microglial cells [23], and heme-oxygenase-1 (HO-1) [24,25], a renowned protein related to antioxidant and anti-inflammatory events that promotes the downregulation of inflammatory markers, were investigated. As depicted in Figure 4A,B, GlycoZIF treatment did not modulate the expression levels of Arginase-1. The absence of Arginase-1 induction reveals that the M2 response is not activated by the presence of GlycoZIF at the working concentration (500 nM). Conversely, the HO-1 protein expression levels were upregulated in response to GlycoZIF regardless of the presence or absence of LPS stimulation (Figure 4A–C). Accumulating evidence discloses the beneficial role played by HO-1 in a variety of inflammatory-related disorders [26]. Its activation contributes to delaying detected cell damage, alleviating inflammation, and regulating different intracellular pathways to counteract the inflammatory signal, being considered a valuable target for treating diabetic complications [27]. In any case, Glyco did not elicit any remarkable effect at the administered dose. However, upregulation of this antioxidant enzyme was also triggered when Bv.2 cells were exposed to ZIF due to the antioxidant [18] and the anti-inflammatory [28] properties of Zn^2+^ ions, which allows us to infer that, in our experimental in vitro inflammatory model, the nanocarrier does not merely behave as a passive vehicle, but also contributes to the activation of HO-1. Although nanosized MOFs are mainly considered passive vehicles in most biomedical applications [29], recently, some authors have paid more attention to the intrinsic immunomodulatory properties of some MOFs, attending to the distinctive bioactivities of their constituents (organic ligands and/or metal ions) [30]. In this line, Horcajada et al. [31] deciphered the immunogenic fingerprint of various nanosized MOFs based on different metal ions (Fe^3+^, Al^3+^, and Zn^2+^), which displayed induction of T-*helper* 1 (Th1) immune response by triggering the secretion of some proinflammatory cytokines.

### 3.4. GlycoZIF Regulates NLRP3 Inflammasome Complex Activity

The inflammasome, a multiprotein complex, plays a crucial role as a modulator of inflammation, so its dysregulation is associated with pathological processes that eventually lead to inflammatory disorders [32]. In fact, therapies targeting inflammasome inhibition have aroused particular interest in the last years. The molecular signaling complex NLRP3 is one of the most widely characterized [33]. Increased HO-1 production is critical for maintaining redox homeostasis and balance [34] and blocking reactive oxygen species (ROS) in the upregulation of NLRP3 production mediated by the transcription factor nuclear factor-κB. This effect on the redox balance has been shown to contribute to the inhibition of NLRP3 activation [35].

**Figure 5:**
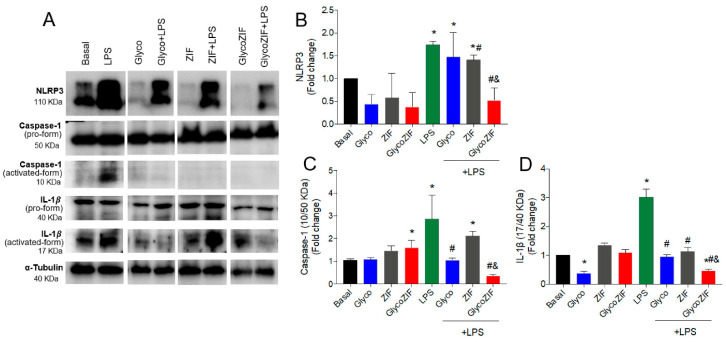
(**A**) Western blot analyses of proteins extracted from Bv.2 microglial cells subjected to the treatment protocol depicted in Figure 3A. Quantification of (**B**) NLRP3, (**C**) Caspase-1, and (**D**) IL-1β. Activation rate in processed proteins such as caspase-1 and IL-1β is represented as the ratio between activated form and pro-form. α-Tubulin was used as a loading control. Results are expressed as mean ± SD (n = 4 independent experiments) and presented as fold-change values relative to untreated control cells (basal value). * *p* ≤ 0.05 vs. basal treatment, ^#^ *p* ≤ 0.05 vs. LPS treatment, ^&^ *p* ≤ 0.05 vs. Glyco + LPS treatment.

Keeping in mind the observed effect of the GlycoZIF on increasing the HO-1 levels, we next investigated the impact of GlycoZIF against LPS-mediated activation of the NLRP3 inflammasome. As observed by Western blot analysis (Figure 5A), a marked dysregulation of LPS-induced NLRP3 protein levels was significantly downregulated after treatment with GlycoZIF particles (Figure 5A,B). It is well known that the inflammatory caspase-1 is cleaved into its active form by activation of the NLRP3 inflammasome, which results in the subsequent catalysis of pro-IL-1β into mature IL-1β, leading to cell lysis and the release of inflammatory factors [36]. As expected, a significant blockage of caspase-1 activation under an inflammatory environment was detected in the presence of GlycoZIF at 500 nM concentration (Figure 5A–C). In quantitative terms, GlycoZIF yielded a 0.54-, 0.24-, and 0.15-fold reduction in NLRP3, caspase-1, and IL-1β, respectively. Again, it is worth highlighting the additive effect observed between Glyco and ZIF to induce a protective effect blocking the cleavage and activation of caspase-1, making the inhibitory effect exerted by GlycoZIF much more pronounced. This set of results clearly reveals the effect of GlycoZIF in the regulation of IL-1β signaling by inhibiting its processing into the mature form (Figure 5A–D).

### 3.5. GlycoZIF Modulates Neuroinflammation in Retinal Explants from BB Rats

**Figure 6:**
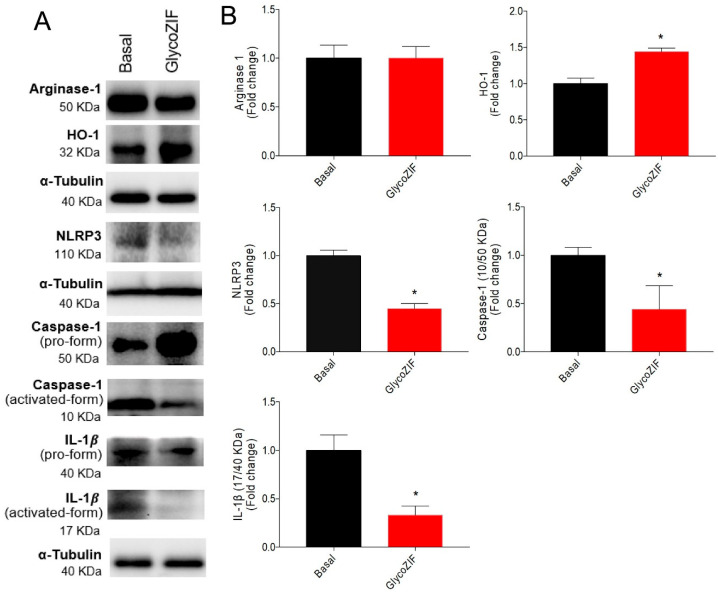
Ex vivo studies with GlycoZIF nanoparticles. (**A**) Western blot analyses of proteins extracted from retinal explants of BB rats at 7 weeks old treated with GlycoZIF (500 nM, 24 h). (**B**) Quantification of the levels of Arginase-1, HO-1, NLRP3, caspase-1, and IL-1β. Activation rate in processed proteins such as caspase-1 and IL-1β is represented as the ratio between the activated form and pro-form. α-Tubulin was used as a loading control. Results are expressed as mean ± SD (n = 4 independent experiments) and presented as fold-change values relative to untreated control cells (basal value). * *p* ≤ 0.05 vs. basal condition.

In light of the great achievements in the in vitro studies, we set out to explore the performance of the GlycoZIF nanoparticles in a physiological retinal model system. For that we used BB rats, a useful animal model for the study of DR featuring retinal pathophysiological processes similar to those found during the progression of human DR, such as inflammation, oxidative stress, and neurodegeneration. Indeed, this animal model is being widely used in preclinical studies for new diabetic inflammatory therapeutic approaches [37]. Retinal explants or organotypic cultures from BB rats provide a physiologically relevant environment in which to study retinal cells, maintaining cell–cell contacts and microenvironmental conditions; this ex vivo culture has successfully allowed us to characterize physiological processes related to retinal disorders [38]. As demonstrated by our previous works, retinal explants from BB rats in an early stage of DR progression (rats at 7 weeks old) maintain the inflammatory events that characterize DR with increased levels of proinflammatory markers [9,39]. We found that the treatment of the retinal explants with GlycoZIF particles (500 nM) promoted the induction of HO-1 expression levels in the retina, consistent with the in vitro results, as well as downregulated the NLRP3 complex with the consequent inhibition of the caspase-1 and IL-1β signaling pathways (Figure 6A,B). These data support that the intracellular mechanisms of action of the GlycoZIF in the retinal explants is the same as that observed in the Bv.2 microglia cells.

**Figure 7:**
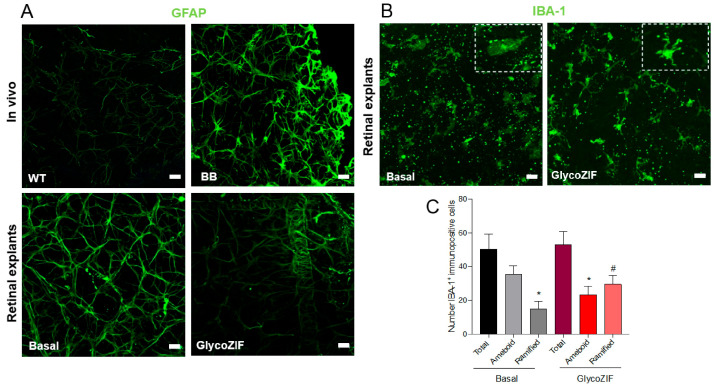
Analysis of inflammatory markers in retinas from BB rats during DR progression. (**A**) GFAP (green) immunostaining in retinal explants from in vivo WT and BB rats or retinal explants at 7 weeks old treated with GlycoZIF (500 nM, 24 h). (**B**) IBA-1 (green) immunostaining in retinal explants from BB rats at 7 weeks old treated with GlycoZIF (500 nM, 24 h). Scale bar: 20 μm. Zoom images (dashed squares) are shown in the inset top right to clearly see the change in the specific phenotypic shape induced by GlycoZIF treatment. (**C**) Immunostaining and quantification of IBA-1^+^ positive cells (ramified or ameboid) in retinal explants from BB rats in basal condition or GlycoZIF treatment. * *p* ≤ 0.05 vs. IBA-1^+^ ameboid basal condition, ^#^ *p* ≤ 0.05 vs. IBA-1^+^ ramified basal condition (n = 5 retinas per condition).

To go a step further, we decided to investigate the impact of GlycoZIF on retinal neuroinflammation, as the progression of T1DM is known to be concurrent with the development of neuroinflammatory events. Specifically, reactive gliosis (i.e., hypertrophy of glial cells) is detected in the retina starting in the early stages of the disease [9,11]. Glial fibrillary acidic protein (GFAP) immunostaining (an upregulated marker of reactive gliosis) was highly expressed in retinal explants from BB rats at 7 weeks old compared with wild-type (WT) rats of the same age, as clearly seen in Figure 7A. Retinal explants from BB rats in the basal condition maintained the GFAP expression, but importantly, we observed that this immunostaining signal was significantly downregulated after 24 h treatment of the retinal explants with GlycoZIF, even at the low working concentration in the nanomolar range (500 nM).

Furthermore, retinal immune cells can display specific phenotypes depending on the cytokine environment to which they are exposed. The healthy retina presents non-activated ramified-shape microglial cells, which change to activated and amoeboid-shaped microglia in response to different stimuli releasing specific patterns of cytokines [40]. Whereas the retinal explants from BB rats showed ameboid microglial cells as expected, upon GlycoZIF treatment, there was a shape switch toward a non-activated state (ramified microglia), which could be explained by the inhibition of key mediators of the inflammatory response owing to the GlycoZIF activity (Figure 7B). The quantification of immunopositive cells of microglial cells (ionized calcium-binding adaptor molecule-1, IBA-1^+^) revealed a higher number of ameboid microglial cells in retinal explants from basal condition compared with those quantified after GlycoZIF treatment. However, the number of ramified microglial cells detected in retinal explants from BB rats exposed to GlycoZIF was markedly increased (Figure 7C). Such a shift toward ramified microglia (i.e., non-active and resting phenotype) indicates a change in the microglia response, specifically for restoring retinal homeostasis, as a result of the inhibition of cytokines production mediated by the GlycoZIF treatment.

## 4. Conclusions

The successful encapsulation of a DR-targeted bioactive glycolipid in biocompatible ZIF-8-type nanostructures (GlycoZIF), together with the intrinsic immunoactive role of the ZIF carrier, has enabled us to efficiently reduce the administered drug dosage in diabetic inflammatory models, minimizing the potential side effects associated with its systemic biodistribution. The dataset obtained from retinal immune cells, and subsequently corroborated in diabetic retinas, validates the outstanding anti-inflammatory activity of GlycoZIF nanoparticles at nanomolar concentrations. The marked increase in the antioxidant enzyme HO-1 mediated by GlycoZIF, which triggers inhibition of the NLRP3 inflammasome pathway, contributes to alleviating the proinflammatory environment, reducing reactive gliosis, and shifting microglia polarization toward a non-activated phenotype. GlycoZIF treatment represents a novel and feasible therapeutic approach that prevents early inflammatory events involved in DR progression, as demonstrated by in vitro and ex vivo assays. Preclinical studies of this GlycoZIF nanosystem in in vivo models of DR due to diabetes mellitus type 1 (BB rat) and type 2 (*db/db* mouse) using a topical ocular administration route (eye drops) are currently being implemented in our laboratories. The knowledge acquired by the encapsulation of different immunomodulatory glycodrugs in nanosized MOF-based structures is expected to provide a solid foundation for treating not only DR, but also other similar ocular diseases caused by diabetes, e.g., retinitis pigmentosa, age-related macular degeneration, etc., as well as other pathologies associated with immune system-mediated inflammation and oxidative stress.

## Data Availability

The original contributions presented in this study are included in the article/Supplementary Materials. Further inquiries can be directed to the corresponding authors.

## Supplementary Materials

The following supporting information can be downloaded at: https://www.mdpi.com/article/10.3390/pharmaceutics17060791/s1. Table S1. List of antibodies; Table S2. List of rat primers; Table S3. Textural properties of GlycoZIF and control ZIF particles; Figure S1. Representative TEM images; Figure S2. ^1^H NMR spectra (400 MHz, CD_3_OD); Figure S3. Flow cytometric analysis of the uptake of FA@ZIF and Confocal microscopy images of Bv.2 cells incubated with FA@ZIF; Figure S4. Inhibitory effect of Glyco, ZIF, and GlycoZIF on *Il1b* and *Il6* mRNA expression levels.

## Abbreviations

The following abbreviations are used in this manuscript:

ARVO	Association for research in vision and ophthalmology
BB	Bio-Breeding
DDS	Drug delivery system
d_h_	Hydrodynamic diameter
DM	Diabetes mellitus
DR	Diabetic retinopathy
DSO_2_-ONJ	(1*R*)-1-Dodecylsulfonyl-5*N*,6*O*-oxomethylidenenojirimycin
EE	Encapsulation efficiency
FA@GlycoZIF	GlycoZIF labeled with fluoresceinamine
FA@ZIF	Control ZIF labeled
FA	Fluorescent cargo
FDA	Food and Drug Administration
FELASA	Federation of European Laboratory Animal Science Associations
GFAP	Glial fibrillary acidic protein
HLPC	High-performance liquid chromatography
HmIM	2-Methylimidazole
HO-1	Heme-oxygenase-1
IBA-1^+^	Ionized calcium-binding adaptor molecule-1
LC	Loading capacity
MAPK	Mitogen-activated protein kinase
MeOH	Methanol
MFI	Mean fluorescence intensity per cell
MOFs	Metal–Organic Frameworks
NJ	Nojirimycin
ROS	Reactive oxygen species
sp^2^-IGLs	sp^2^-Iminoglycolipids
T1DM	Type 1 DM
Th1	T-*helper* 1
T-PBS	Tween-20-PBS
VEGF	Vascular endothelial growth factor
WT	Wild type
ZIF	Zeolitic-Imidazolate Framework
ZIF-8	Zeolitic Imidazolate Framework-8
