# Supplementary material for: Exploring a Novel Anti-Inflammatory Therapy for Diabetic Retinopathy Based on Glyco-Zeolitic-Imidazolate Frameworks
Source: Pharmaceutics. 2025 Jun 17;17(6):791. doi: 10.3390/pharmaceutics17060791 (PMC12196511; doi:10.3390/pharmaceutics17060791)
Supplement: Supplementary file 1 [file pharmaceutics-17-00791-s001.zip › pharmaceutics-3665742-supplementary.pdf]

SUPPLEMENTARY INFORMATION

Exploring a Novel Anti-inflammatory Therapy for Diabetic Retinopathy Based on Glyco-Zeolitic-Imidazolate Frameworks

Elena Díaz-Paredes [1,†], Francisco Martín-Loro [1,†], Rocío Rodríguez-Marín [2], Laura Gómez-Jaramillo [1], Elena M. Sánchez-Fernández [2,]*, Carolina Carrillo-Carrión [3,]* and Ana I. Arroba [1]

[1] Department of Endocrinology, INiBICA, Puerta del Mar University Hospital, University of Cádiz, Avda. Ana de Viya 21, 11009 Cádiz, Spain

[2] Department of Organic Chemistry, Faculty of Chemistry, University of Sevilla, C/ Profesor García González 1, 41012 Sevilla, Spain

[3] Institute for Chemical Research (IIQ), CSIC—University of Seville, Avda. Américo Vespucio 49, 41092 Sevilla, Spain

* Correspondence: esanchez4@us.es (E.M.S.-F.), carolina.carrillo@csic.es (C.C.-C.)

[†] These authors contributed equally to this work.

Table S1. List of Antibodies.

Antibodies	Company	Reference	Technical Approach	Working Concentration
α-Tubulin	Sigma-Aldrich (St. Louis, USA)	T5168	WB	1:10000
HO-1	Abcam (Cambridge, UK)	AB189491	WB	1:1000
Caspase-1	Abcam (Cambridge, UK)	AB179515	WB	1:1000
IL-1β	Mybiosurce (San Diego, USA)	MBS821750	WB	1:1000
NLRP3	AdipoGene Life Sciences (Füllinsdorf, Switzerland)	AG-20B-0014-C100	WB	1:1000
Rabbit-Peroxidase	Sigma-Aldrich (St. Louis, USA)	A0545	WB	1:5000
Mouse-Peroxidase	Sigma-Aldrich (St. Louis, USA)	A2554	WB	1:5000
Arginase-1	BD Biosciences (Madrid, Spain)	610708	IF/WB	1:400/1:1000
GFAP	DAKO (Glostrup, Denmark)	Z0334	IF	1:500
IBA-1	Fujifilm-Wako (Madison, USA)	019-19741	IF	1:500
Rabbit Alexafluor-488	Thermo Fisher (Waltham, USA)	A11034	IF	1:1000

Table S2. List of rat primers.

Gene		*Mus musculus*
Gapdh	Forward	CGCTGTATTCCCCTCCATCG
	Reverse	CCAGTTGGTAACAATGCCATGT
Tnfa	Forward	CCCTCACACTCAGATCATCTTCT
	Reverse	GCTACGACGTGGGCTACAG
Il1b	Forward	GCAACTGTTCCTGAACTCAACT
	Reverse	ATCTTTTGGGGTCCGTCAACT
Il6	Forward	TACCACTTCACAAGTCGGAGGC
	Reverse	CTGCAAGTGCATCATCGTTGTTC

Table S3. Textural properties of GlycoZIF and control ZIF particles.

Sample	S_{BET} (m^2/g)	S_{micro} (m^2/g)	S_{ext} (m^2/g)	V_{micro} (cm^3/g)
GlycoZIF	1194	946	248	0.435
ZIF	1243	1213	29	0.565

Total surface area calculated by BET equation (S_{BET}); micropore area (S_{micro}), external surface area (S_{ext}) and micropore volume (V_{micro}) calculated by t-plot method.

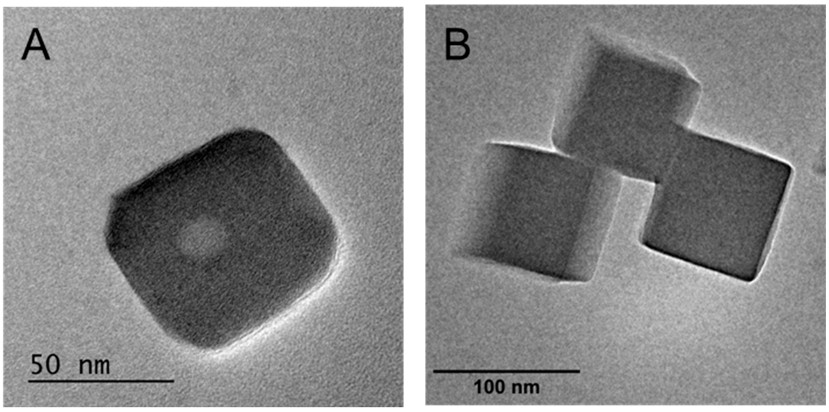

Figure S1. Representative TEM images of the (A) GlycoZIF and (B) control ZIF particles.

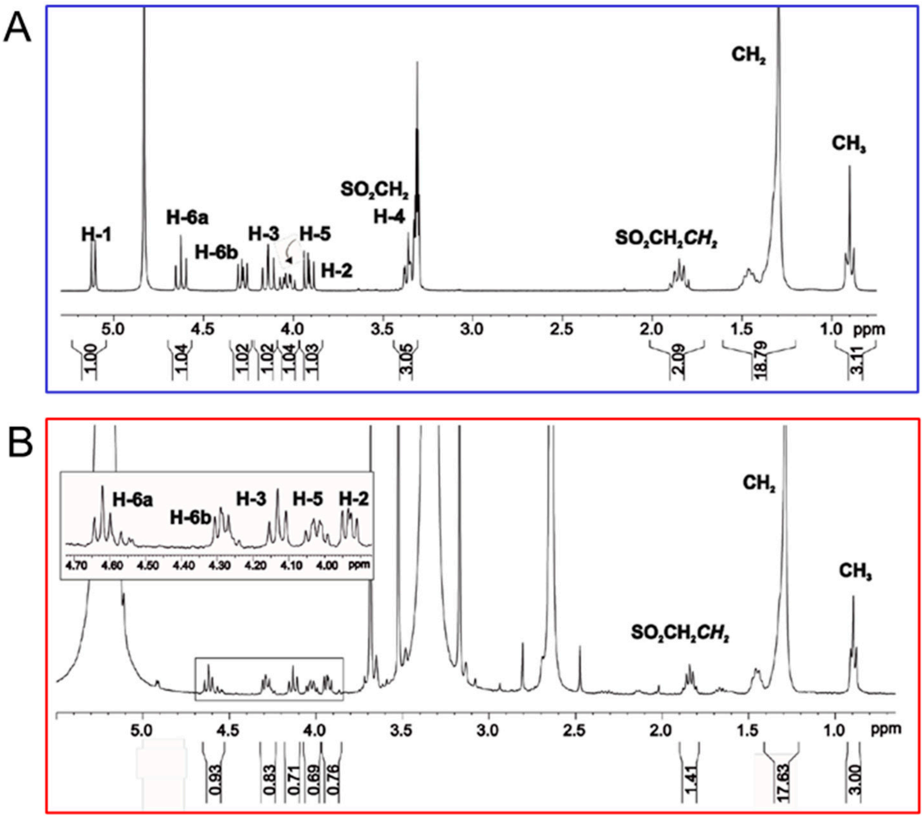

Figure S2. ^{1}H NMR spectra (400 MHz, CD_3OD) of (A) the pure Glycolipid as reference, and (B) the mixture after acid dissolution of the GlycoZIF.

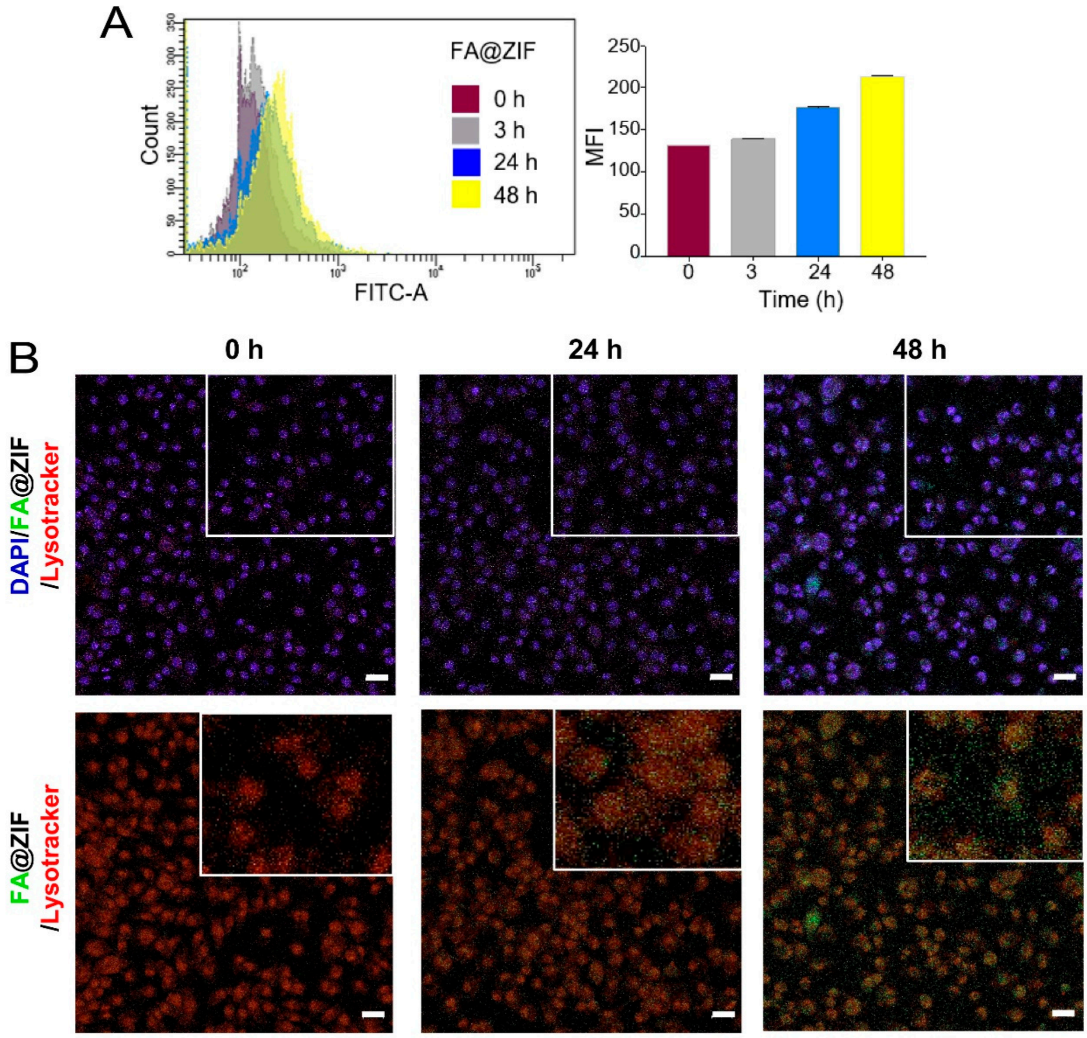

Figure S3. (A) Flow cytometric analysis of the uptake of FA@ZIF (500 nM) by Bv.2 cells, showing the histograms and the corresponding mean fluorescence intensity (MFI) values as a function of the exposure time (0 h, 3 h, 24 h and 48 h). (B) Confocal microscopy images of Bv.2 cells incubated with FA@ZIF (500 nM) for different exposure times (0 h, 24 h and 48 h). Green fluorescence corresponds to the encapsulated cargo (*i.e.*, FA), while red fluorescence corresponds to LysoTracker and blue fluorescence to the stained nucleus. Higher magnifications (white square) are shown in the inset top right. Scale bar: 20 µm.

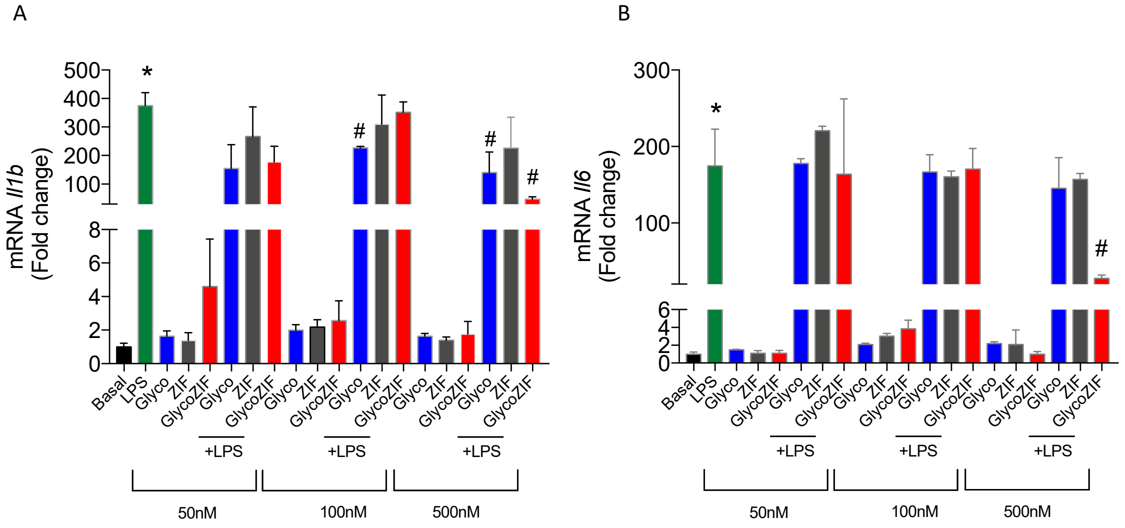

Figure S4. Inhibitory effect of Glyco, ZIF and GlycoZIF on *Il1b* (A) and *Il6* (B) mRNA expression levels at different doses in LPS-stimulated Bv.2 microglial cells. Data were normalized to *Gapdh* gene expression. Results are expressed as mean ± SD (n = 5 independent experiments) and presented as fold-change values relative to untreated control cells (basal value). * $p \leq 0.05$ vs. basal treatment, # $p \leq 0.05$ vs. LPS treatment.
